# Low Cycle Fatigue Life Prediction Using Unified Mechanics Theory in Ti-6Al-4V Alloys

**DOI:** 10.3390/e22010024

**Published:** 2019-12-23

**Authors:** Noushad Bin Jamal M, Aman Kumar, Chebolu Lakshmana Rao, Cemal Basaran

**Affiliations:** 1 Department of Applied Mechanics, Indian Institute of Technology, Madras 600036, India; noushadbj@gmail.com (N.B.J.M.); kumaraman2102@gmail.com (A.K.); lakshman@iitm.ac.in (C.L.R.); 2 Civil, Structural and Environmental Engineering, University at Buffalo, State University of New York, New York, NY 10031, USA

**Keywords:** entropy, fatigue, damage mechanics, unified mechanics, thermodynamics, Ti-6Al-4V, physics of failure

## Abstract

Fatigue in any material is a result of continuous irreversible degradation process. Traditionally, fatigue life is predicted by extrapolating experimentally curve fitted empirical models. In the current study, unified mechanics theory is used to predict life of Ti-6Al-4V under monotonic tensile, compressive and cyclic load conditions. The unified mechanics theory is used to derive a constitutive model for fatigue life prediction using a three-dimensional computational model. The proposed analytical and computational models have been used to predict the low cycle fatigue life of Ti-6Al-4V alloys. It is shown that the unified mechanics theory can be used to predict fatigue life of Ti-6Al-4V alloys by using simple predictive models that are based on fundamental equation of the material, which is based on thermodynamics associated with degradation of materials.

## 1. Introduction

Titanium alloys are popular for their superior mechanical properties, such as high yield strength, long fatigue life, toughness, low density, as well as corrosion resistance. About 80% of the global production of titanium alloys are used by aerospace industries [1]. One of the widely used titanium alloys is Ti-6Al-4V [2] which has a dual-phase crystal structure, namely, hexagonal close packed (HCP) and body centered cubic (BCC) structures. In the composition of Ti-6Al-4V alloy, titanium is the matrix material. Aluminium plays the role of stabilizing the HCP structure and vanadium preserve the BCC structure [3]. Many applications of Ti-6Al-4V alloys, such as aero engines, are subjected to cyclic loading [4]. Hence, it is essential to predict the fatigue life of such structural components, when they are subjected to varying amplitudes of cyclic loading during their service period. It is not always feasible to conduct fatigue experiments corresponding to all service conditions. Hence, predictive models based on fundamental physics of materials are helpful in predicting the fatigue life of structures.

A number of studies have been published to investigate the fatigue life of metals. Most of the damage prediction models are based on statistical test data analysis or on experimental curve fit [5,6,7,8,9,10,11]. Low cycle fatigue life prediction in Ti-6Al-4V alloys are generally done, based on stress [12], strain [5,6,13,14,15,16] or hysteresis loss [17]. Most of them are empirical curve-fit models [7,9,13,18,19,20,21,22] or mechanism based phenomenological models [23,24,25] such as fatigue crack initiation models [16]. A detailed review of such models, applied to metals, can be seen in the review article by Santecchia et al. [26]. A model, based on combined Newtonian mechanics and thermodynamics, instead of material-specific and loading-specific, can capture the mechanisms of fatigue damage without the need for curve fitting process. 

If the system is less complicated and we want a quick solution we can opt for a one-dimensional model based on certain assumptions. However, validity of the model depends upon the accuracy of the assumptions made while formulation of one-dimensional analytical model. The interpretation of the results using one-dimensional model is also easy as it can be simple in its form and usage. A number of one-dimensional empirical curve-fit fatigue life prediction models can be seen in the literature [5,6,7,8,11,12,14,15,16,17]. Nevertheless, a physics-based one-dimensional model, which can be easily used to predict the fatigue life of Ti-6Al-4V, under appropriate assumptions, is still not found in the literature. If the system is very complicated to arrive at suitable one-dimensional fatigue life prediction model, we look for another appropriate and convenient method. It is known that, a three dimensional computational model can be incorporated with appropriate material nonlinearities (such as plastic flow), to account for the experimental observations [10,22] and to limit the assumptions in developing the model. However, a large number of cyclic loading simulation in a three dimensional numerical model is computationally very expensive [10]. Hence, it is very useful to have an appropriate physics-based procedure, in conjunction with three-dimensional numerical results, to account for all the nonlinearities associated with the computational model, even as we maintain the simplistic predictive capability of a one-dimensional model. Therefore, the present study is focused on both one-dimensional and three-dimensional, thermodynamics-based modeling of the deformation of standard test specimen to predict the fatigue life of Ti-6Al-4V.

Thermodynamics is a field of science that is developed to study change in the state of matter. The historical development of thermodynamics from its classical form to modern-age form has been reviewed by Haddad et al. [27,28]. Between 1872 and 1875, Boltzmann gave a mathematical expression to second law of thermodynamics for quantification of order/disorder in terms of a measure called *entropy*. In 1998, Basaran and Yan [29] introduced the unified mechanics theory, which unifies Newtonian mechanics with thermodynamics. In unified mechanics theory [29], in addition to nodal displacements, the entropy generation rate is also necessary to relate microstructural changes in the material with spatial and temporal coordinates. This concept [29] has been successfully implemented for a wide range of materials and has been experimentally and mathematically validated and reported in literature [18,19,20,25,30,31,32,33,34,35,36,37,38,39,40,41,42,43,44,45,46,47,48,49,50,51,52,53,54,55,56,57,58,59,60,61,62,63,64,65]. The entropy generation rate of any material under any external disturbances like mechanical, thermal, electrical, chemical, radiation, and corrosion can be calculated from principles of physics, using the fundamental equation, with no need for curve fitting phenomenological models or polynomials fit to experimental test data. 

In the present study, unified mechanics theory is used to estimate the fatigue damage in Ti-6Al-4V, analytically with a one -dimensional (1-D) model as well as numerically with a three-dimensional (3-D) model, and this damage estimation procedure has been used to predict fatigue life under different loading conditions. Fundamental details of the unified mechanics theory-based fatigue life prediction are summarized in Section 2. The principles described in Section 2, are then applied to Ti-6Al-4V, by considering the plasticity as the dominant energy dissipation mechanism.

In order to establish the validity of the proposed model in cyclic loading, comparison of simulation with experimental results, under both the tensile and compressive loading are necessary. In Section 3, the details of implementation and validation of the proposed model, for both compressive and tensile monotonic loading is presented. After the validation of the proposed model, we introduce two different procedures, to estimate the low cycle fatigue life of Ti-6Al-4V alloys in Section 4. Finally, the observations from the presented work are discussed in Section 5, based on the observations made on the principles, procedure and results from the current study for the fatigue life prediction of Ti-6Al-4V alloys.

## 2. Unified Mechanics Theory-Based Life Prediction Model

### 2.1. Unified Mechanics Theory

Unified mechanics theory is just unification of Newton’s universal laws of motion and laws of thermodynamics.

#### 2.1.1. Second Law of Unified Mechanics Theory

Initial momentum of a mass, *m*, subjected to external force, **F** is defined by Newton’s second universal law of motion. However, Newton’s laws do not account for energy loss after the initial momentum. Energy loss takes place according to the first and second laws of thermodynamics. As a result, a marriage of laws of second law of Newton and laws of thermodynamic is given by:

(1) $$F=\frac{dP}{dt}=\frac{d\left(mv\right)}{dt}\left(1-\Phi \right)$$

where, **P** represents the momentum and v represents the velocity. Assuming a constant mass system,

(2) $$F=m\frac{d\left[v\left(1-\Phi \right)\right]}{dt}$$

where, Φ is the Thermodynamic State Index (TSI), which is normalized non-dimensional form of the second law of thermodynamics. TSI (Φ) starts at zero and reaches one when the system reaches maximum entropy and minimum entropy generation rate. The value of TSI (Φ) is calculated from the fundamental equation of the material, which accounts for all entropy generation mechanisms in the system under the given load towards a pre-defined failure. The fundamental equation must satisfy the conservation of energy, the first law of thermodynamics at every step. Therefore, TSI (Φ) just introduces laws of thermodynamics in to the laws of Newton.

#### 2.1.2. Third Law of Unified Mechanics Theory 

All forces between two objects exist in equal magnitude and opposite direction (Action–Reaction). However, resulting deformation, according to Hook’s law, in two objects will change over time because of degradation. The resulting equation can be given by:

(3) $$F_{12}=F_{21}\left[1-\Phi \right]$$

where, the subscripts 12 and 21 represents the action and reaction, respectively. Based on Hooke’s law, the reaction, $F_{21}$ can be given by the following:

(4) $$F_{12}=\frac{dU_{21}}{du_{21}}=\frac{\left[d\left[\frac{1}{2}\;k_{21}\left[1-\Phi \right]\;u_{21}^{2}\right]\right]}{du_{21}}$$

where, $U_{21}$ is the strain energy of the reactionary member, $k_{21}$ is the stiffness of the reactionary member, $u_{21}$ is the displacement in the reactionary member. If we assume that for the increment of displacement, $du_{21}$ derivative of TSI with respect to $du_{21}$ is smaller than derivative of displacement $u_{12}$ by an order of magnitude as the differential in displacement $du_{21}$ goes to zero in the limiting case, we can write the following simple relation:

(5) $$F_{12}=k_{21}\left[1-\Phi \right]\;u_{21}$$

In unified mechanics theory, it has been shown that the degradation of the stiffness follows the laws of thermodynamics [8,18,20,22,27,29,30,31,32,33,35,36,37,38,39,40,41,42,43,44,45,46,47,48,49,50,51,52,53,54,56,57,58,59,66,67,68,69]. Combining laws of Newton and thermodynamics requires the modification of Newtonian space-time coordinate system. A new thermodynamic axis must be added to be able to define the thermodynamic state of a point. As a result, the motion of any particle can be defined only in a five-dimensional space that has five linearly independent axes. None of these axes can represent the information of other axes. Hence, entropy generation can be mapped onto a non-dimensional coordinate called Thermodynamics State Index (TSI) which is necessary to locate the thermodynamic state of the particle. Coordinates of a point can be defined by Newton’s laws of motion in the space-time coordinate system. However, thermodynamic state coordinate cannot be defined by space-time coordinate system. 

**Figure 1:**
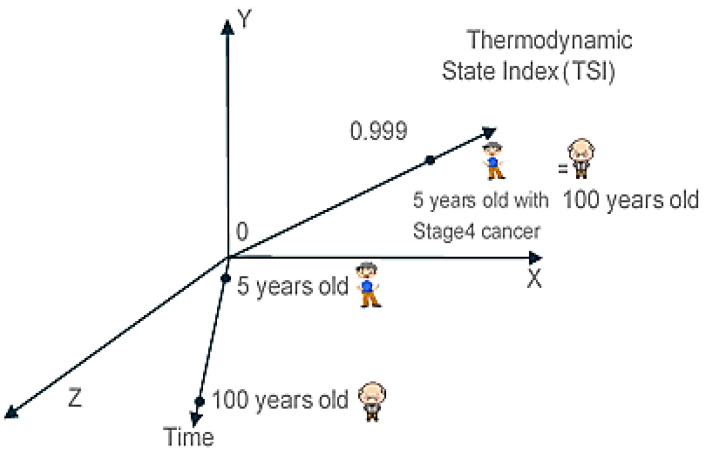
Coordinate system in unified mechanics theory.

Figure 1 shows the coordinate system in unified mechanics theory. Let us assume there is a 5-year-old boy and 100-year-old man. Using the space-time Cartesian coordinate system, their location can be defined by x,y,z coordinates and age on the time axis. However, this does not give any information about their thermodynamic state. Let us assume that a 5-year-old boy has stage 4 cancer is expected to die in a few days and a 100-year-old is expected to die in few days. This information cannot be represented in x,y,z-time- space coordinate system as shown in Figure 1. However, on TSI axis, 5-year-old boy and 100-year-old will have the same thermodynamic state index coordinate at Φ=0.999.

Another example can be given for Newton’s second law. If a soccer ball is given an initial acceleration with a force of F, it will move but eventually will come to a stop. Depending on the path it follows, it will come to a stop. Again, the initial acceleration of the ball is governed by the second law of Newton and slowing down process is governed by the laws of thermodynamics, which is represented by (1−Φ) term. Detailed derivation of TSI can be seen in the literature [29]. We provide a simple summary in the following section.

#### 2.1.3. Thermodynamic State Index (TSI) for Damage in Low Cycle Fatigue of Materials

Entropy and Helmholtz free energy are related by the thermodynamic principles [66] as follows:

(6) Ψ=e−Ts

where Ψ represents the specific Helmholtz free energy, and *e*, *T*, *s* are the specific internal energy, temperature and specific entropy, respectively. Specific entropy is also related to the disorder parameter through Boltzmann’s equation [29,30] as follows:

(7) $$s=k_{B}\ln\left(W\right)$$

Total entropy for a volume can be given by:

(8) $$S=\frac{N_{A}k_{B}\;ln\left(W\right)}{m_{s}}$$

where, $N_{A}$, $k_{B}$, $m_{s}$ are the Avogadro number, Boltzmann’s constant and molar mass, respectively and W represent the disorder parameter [29,30,38,39,66]. Relation between the number of microstate, probability of microstates and disorder parameter is discussed extensively in the literature [70,71,72]. Using Equation (8), the TSI is given by:

(9) $$\Phi =\Phi_{c}\left(1-\exp\left(-\Delta s\frac{m_{s}}{R}\right)\right)$$

where, $\Phi_{c}$, is a user defined parameter, representing the predefined failure criterion. R is gas constant. Δs is a measure of the total change in entropy at a point. Unified mechanics theory states that when a system undergoes thermodynamic change from state A to state B, the remaining useful life can be defined by a factor in each stage of its life, called thermodynamic state index (TSI), Φ∈ [0,1]. The ultimate failure is represented by a value of TSI equal to 1. Since, the value of Δs is to be evaluated on the basis of mechanisms of dissipation processes involved in a thermodynamic process, the value of $\Phi_{c}$ will be governed by a user-defined ultimate failure criterion. 

### 2.2. Analytical Approach for the Prediction of Damage and Fatigue Life

**Figure 2:**
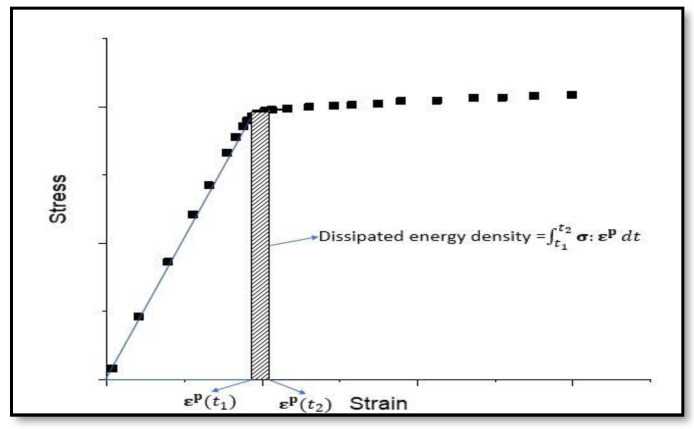
Schematic representation of computing plastic dissipation from the engineering stress-plastic strain graph.

From Equation (9), the TSI is governed by the change in entropy towards a predefined failure. All the dissipation processes that are related to failure lead to increase in entropy. Therefore, an appropriate measure of dissipation is needed to estimate the life of a process. In Ti-6Al-4V alloys, we consider only the mechanical process of dissipation, under monotonic as well as cyclic loading conditions. Hence the plastic dissipation is considered to be the dominant mechanism in the mechanical loading conditions. Entropy generation in plastic dissipation process can be calculated from a mechanical loading experiment in the following way:

(10) $$\Delta s=\frac{1}{\rho T}\int_{t_{1}}^{t_{2}}\sigma :d\varepsilon^{p}$$

where, ρ, is the mass density of the material, σ and $\varepsilon^{p}$ are the stress and plastic strain, respectively. *T* represents the temperature. Integral limits $t_{1}$ and $t_{2}$ represents the time bounds of the mechanical loading process, over which we quantify the change in entropy. For one dimensional case, the total plastic strain, $\varepsilon^{p}\left(t\right)$ is calculated as follows:

(11) $$\varepsilon^{p}\left(t\right)=\varepsilon^{total}\left(t\right)-\frac{\sigma_{y_{0}}}{E}$$

where, $\varepsilon^{total}\left(t\right)$ is the total strain at the time of loading, *t*. $\sigma_{y_{0}}$ and E are the yield stress and Young’s modulus, respectively. In the case of monotonic loading, the plastic dissipation is calculated from the engineering stress-strain graphs. In order to accomplish this, the plot is divided into elastic and plastic regime of loading. The area under the plastic region is computed by trapezoidal integration rule, and the cumulative entropy is evaluated in each stage. This accumulated entropy is used to predict the TSI at each and every strain level. A schematic representation of computing the incremental plastic dissipation is given in Figure 2. Accumulated entropy at *n*-th strain increment is computed from the Equation (10) as follows:

(12) $$\Delta s_{n}=\frac{1}{\rho T}\overset{i=n}{\underset{i=1}{\sum }}\sigma_{i}:\Delta \varepsilon_{i}^{p}$$

Using Equations (9), (11) and (12), one dimensional approximation of damage measure is calculated under the assumptions that the damage is uniform within the cross section of the dog-bone test sample, and there are no other geometric or boundary effects in the sample. It is also assumed that the heat generation entropy production is small when compared with the entropy generation due to plastic deformation. In case of low cycle fatigue loading, the plastic dissipation is calculated as the area under the stress-strain hysteresis loop. Each cyclic hysteresis loop of engineering stress-strain graph, which represents the incremental dissipation. Hence, the accumulated entropy can be calculated by summation of incremental entropy. For a strain-controlled experiment, the accumulated entropy is a function of stress. Since the stress level at a given stage of cyclic loading is governed by the thermodynamic state index (TSI), Φ of the material, the TSI can be used to calculate the incremental dissipation from any known stage of loading, as follows:

(13) $$\Pi_{i+1}^{p}=\left(1-\Phi_{i}\right)\Pi_{i}^{p}$$

where, $\Pi_{i}^{p}$ and $\Pi_{i+1}^{p}$ represents the hysteresis area at *i*-th and (*i*+1)-th cyclic loading, respectively and Φ represents the TSI. Hence, the entropy change at any loading stage can be calculated from the initial loading hysteresis area as follows:

(14) $$\Delta s_{n}=\frac{1}{\rho T}\overset{i=n}{\underset{i=1}{\sum }}\Pi_{i}^{p}$$

(15) $$\Phi_{i+1}=\Phi_{c}\left[1-\exp\left(-\Delta s_{i}\frac{m_{s}}{R}\right)\right]$$

It is important to point out that the entire thermodynamic response of the material point is mapped onto the TSI axes. Under no circumstances, the material point can exist outside the domain of [0,1]. The above approach has limitations that the one-dimensional approximation should be valid when the prediction is compared with experimental observations. To account for all the boundary and geometric effects related to stiffness, instabilities due to buckling, local cracking, stress concentrations, geometric nonlinearities, etc., we have developed a three-dimensional computational model. The detailed derivation is given in Section 2.3 below.

### 2.3. Computational 3-D Model for the Prediction of Damage 

#### 2.3.1. Derivation of the Computational Model

In this section, a three-dimensional model is derived, based on the unified mechanics theory. Entropy balance equation [4,20,29,30], can be written as follows:

(16) $$\frac{dS}{dt}\geq -\frac{div\;J_{q}}{T}+\frac{1}{T}\sigma :D-\frac{\rho }{T}\frac{dW^{e}}{dt}+\frac{\rho 𝓇}{T}$$

The following equation, as written in indicial notation, is known as Clausius-Duhem inequality [67,73,74]:

(17) $$ɤ=\frac{1}{T}\sigma_{ij}D_{ij}-\frac{\rho }{T}\frac{dW^{e}}{dt}-\frac{1}{T^{2}}J_{q}{}_{i}T_{,i}+\frac{\rho 𝓇}{T}\geq 0$$

where, *i* and *j* are the indices, representing the spatial coordinates. ɤ is the specific entropy generation rate. σ denotes the stress tensor and $T_{,i}$ represents the spatial derivative of temperature, namely, the gradient of temperature. $J_{q}$ and 𝓇, represents the heat flux transfer and internal heat generation, respectively. For small strain problems, rate of deformation tensor D is equal to strain rate tensor $\dot{\varepsilon }$. According to Hooke’s law, stress is related to the strain through a constitutive tensor as follows:

(18) $$\sigma_{ij}=C_{ijkl}\varepsilon_{kl}^{e}$$

where, $C_{ijkl}$ is the fourth order tangential constitutive tensor at the given stage of loading. $\varepsilon_{kl}^{e}$ is the elastic part of strain tensor. Based on assumption of the additive decomposition of strain tensor [67], we can write the following equation for small strain problems:

(19) $$\varepsilon_{ij}^{total}=\varepsilon_{ij}^{e}+\varepsilon_{ij}^{p}$$

where, $\varepsilon_{ij}^{total}$ is the component of total strain tensor. For a given material point, based on unified mechanics theory one can write the following modified version of Equation (18), as follows:

(20) $$\sigma_{ij}=\left(1-\Phi \right)C_{ijkl}^{0}\varepsilon_{kl}^{e}$$

where the tangential constitutive tensor $C_{ijkl}$ is related to the virgin state of the same, $C_{ijkl}^{0}$ (undamaged state) through TSI, Φ. For linear isotropic materials, undamaged constitutive tensor $C_{ijkl}^{0}$ can be written as follows:

(21) $$C_{ijkl}^{0}=\lambda \delta_{ij}\delta_{kl}+\mu \left(\delta_{ik}\delta_{jl}+\delta_{il}\delta_{jk}\right)$$

where, λ and μ are the Lame’s parameters and $\delta_{ij}$ is the identity tensor. The following inverse relations can also be written for a linear elastic isotropic material:

(22) $$\varepsilon_{ij}^{e}=\frac{1+\nu }{E}\sigma_{ij}-\frac{\nu }{E}\sigma_{kk}\delta_{ij}$$

where, *E* and ν are the elastic modulus and Poisson’s ratio, respectively. The rate form of the Equation (19), can be written as follows,

(23) $${\dot{\varepsilon }}_{ij}^{total}={\dot{\varepsilon }}_{ij}^{e}+{\dot{\varepsilon }}_{ij}^{p}$$

From incremental theory of plasticity, one can write the evolution equation for the fluxes, using the continuity of dissipation potential function, ${ℱ}^{p}$ (yield surface) [67] as follows:

(24) $${\dot{\varepsilon }}_{ij}^{p}=\dot{\Gamma }\frac{\partial {ℱ}^{p}}{\partial \sigma_{ij}}$$

Effective stress at a point can be defined as follows:

(25) $$\sigma_{ij}^{\prime }=\frac{1}{1-\Phi }\sigma_{ij}$$

where, $\sigma_{ij}^{\prime }$ is the component of effective stress tensor. Noting that Δs is the only function that depends on time, the time rate of change of TSI can be obtained by differentiating Equation (9), yielding:

(26) $$\dot{\Phi }=\frac{m_{s}}{R}\Phi_{c}\dot{\Delta s}\left(\exp\left(-\Delta s\frac{m_{s}}{R}\right)\right)$$

Assuming that the process is isothermal for each small load increment and all the dissipation mechanisms other than plastic deformation are negligibly small for the strain-controlled monotonic, quasi-static loading and low cycle fatigue loading in Ti-6Al-4V, we can write the entropy evolution as given in Equation (10). Hence the rate form of the entropy evolution from Equation (10) can be written as follows:

(27) $$\dot{\Delta }s=\frac{1}{\rho T}\sigma_{ij}{\dot{\varepsilon }}_{ij}^{p}$$

With the above assumption in the absence of kinematic hardening, we consider the following additive decomposition form of the Helmholtz free energy function as:

(28) $$\Psi \left(\varepsilon^{e},h;\Phi \right)=\Psi^{E}\left(\varepsilon^{e};\Phi \right)+\Psi^{I}\left(h\right)$$

where, $\Psi^{E}$ is the elastic strain energy and $\Psi^{I}$ is the free energy from isotropic hardening process. In the Equation (28), the hardening flux parameter h evolves with plastic strain. From the Equations (26) and (27), the plastic strain is a function of TSI. 

Using Equations (20), (21) and (28), we get the following form of free energy:

(29) $$\Psi \left(\varepsilon^{e},h;\Phi \right)=\frac{1}{2}\left(1-\Phi \right)\left(\lambda \varepsilon_{kk}^{e}\varepsilon_{mm}^{e}+2\mu \varepsilon_{ij}^{e}\varepsilon_{ij}^{e}\right)+\left(1-\Phi \right)\frac{1}{r}Kh^{r+1}$$

We have assumed a power law model for isotropic hardening. Here, *K* and r are the material parameters which are to be found from the succeeding parts of the formulation and experimental data. The conjugate force is derived from Equation (29) as follows [75]:

(30) $$\sigma =\rho \frac{\partial }{\partial \varepsilon^{e}}\Psi$$

(31) $$\sigma_{ij}=\left(1-\Phi \right)\left(\lambda \varepsilon_{kk}^{e}\delta_{ij}+2\mu \varepsilon_{ij}^{e}\right)$$

The yield function for Ti-6Al-4V can be given by:

(32) $${ℱ}^{p}\left(\sigma ,H;\Phi \right)=\sigma_{eq}^{\prime }-\left(\sigma_{y_{o}}+H\right)$$

where, $\sigma_{eq}^{\prime }$ is the Von-Mises equivalent stress. $\sigma_{y_{o}}$ represents the initial yield stress and *H* represents the hardening stress. Von-Mises equivalent stress is given by the following equation:

(33) $$\sigma_{eq}^{\prime }=\sqrt{\frac{3}{2}S_{ij}^{\prime }S_{ij}^{\prime }}$$

where, the effective deviatoric stress tensor $S_{ij}^{\prime }$, is given by the following equation,

(34) $$S_{ij}^{\prime }=\sigma_{ij}^{\prime }-\frac{\sigma_{kk}^{\prime }}{3}\delta_{ij}$$

Hence, from Equations (24) and (32), we get the following relation for plastic strain rate tensor, ${\dot{\varepsilon }}_{ij}^{p}$,:

(35) $${\dot{\varepsilon }}_{ij}^{p}=\dot{\Gamma }\frac{\partial \sigma_{eq}^{\prime }}{\partial \sigma_{ij}}$$

Further simplification can be done on Equation (35) using the Equations (33) and (34). We get the following form for plastic strain rate tensor, based on normality rule of incremental theory of plasticity:

(36) $${\dot{\varepsilon }}_{ij}^{p}=\dot{\Gamma }\left[\frac{1}{\left(1-\Phi \right)}\frac{3}{2}\frac{S_{ij}^{\prime }}{\sigma_{eq}^{\prime }}\right]$$

where, $\dot{\Gamma }$ is the consistency parameter. By taking the norm of Equation (36) and by doing some algebra, we get the following equation to quantify the parameter, $\dot{\Gamma }$:

(37) $${\dot{\varepsilon }}_{eq}^{p}=\sqrt{\frac{2}{3}{\dot{\varepsilon }}_{ij}^{p}{\dot{\varepsilon }}_{ij}^{p}}=\dot{\Gamma }\frac{1}{\left(1-\Phi \right)}$$

Equation (37) is an important observation that the field variable, h, representing the isotropic hardening process, is related to the plastic deformation. Hence, we get the following form for $\dot{h}$ and ${\dot{\varepsilon }}_{ij}^{p}$:

(38) $$\dot{h}={\dot{\varepsilon }}_{eq}^{p}\left(1-\Phi \right)$$

(39) $${\dot{\varepsilon }}_{ij}^{p}={\dot{\varepsilon }}_{eq}^{p}\left[\frac{3}{2}\frac{S_{ij}^{\prime }}{\sigma_{eq}^{\prime }}\right]$$

From Equation (39), it can be observed that the magnitude of plastic strain is given by the equivalent plastic strain, $\varepsilon_{eq}^{p}$, and the direction of plastic loading is given by the term, $\left[\frac{3}{2}\frac{S_{ij}^{\prime }}{\sigma_{eq}^{\prime }}\right]$. 

#### 2.3.2. Algorithm for the Computational Model

In this section, let us consider that all the variables having a superscript, ‘*n*’ represents values that are updated based on the previous loading and those variables with superscript, ‘*n*+1’ denotes the values corresponding to the current state of loading. All the variables having subscript, ‘*tr*’ represents the trial values. For simplicity in representation, indicial representation of the tensorial quantities are avoided.

Total strain at any increment is given by:

(40) $$\varepsilon^{{\mathrm{total}}^{n+1}}=\varepsilon^{{\mathrm{total}}^{n}}+\Delta \varepsilon^{\mathrm{total}}$$

Using Equation (19):

(41) $$\varepsilon^{e}{}_{tr}^{n+1}=\varepsilon^{{\mathrm{total}}^{n+1}}-\varepsilon^{p^{n}}$$

Using Equation (20):

(42) $$\sigma_{tr}^{n+1}=\left(1-\Phi^{n}\right)C^{0}\varepsilon^{e}{}_{tr}^{n+1}$$

(43) $$\sigma^{n+1}=\left(1-\Phi^{n+1}\right)C^{0}\left(\varepsilon^{{\mathrm{total}}^{n+1}}-\varepsilon^{p^{n+1}}\right)$$

Let:

(44) w=(1−Φ)

then:

(45) $$\sigma^{n+1}=w^{n+1}C^{0}\left(\varepsilon^{{\mathrm{total}}^{n+1}}-\varepsilon^{p^{n}}-\Delta \varepsilon^{p}\right)$$

Using Equations (25), (36), (40), (41), and (44) in Equation (45), we get the following:

(46) $${\sigma^{\prime }}^{n+1}=\frac{1}{w^{n}}\sigma_{tr}^{n+1}-\frac{1}{w^{n+1}}C^{0}\Delta \Gamma \left[\frac{3}{2}\frac{{S^{\prime }}^{n+1}}{\sigma_{eq}^{\prime }}\right]$$

Let:

(47) $$p^{\prime }=\frac{\sigma_{kk}^{\prime }}{3}$$

Therefore, from Equations (34), (46) and (47), we can write the following expression:

(48) $${S^{\prime }}^{n+1}+\frac{1}{w^{n}}p_{tr}\;I-\frac{1}{3}C^{0}\frac{\Delta \Gamma }{w^{n+1}}\;\left[\frac{3}{2}\frac{{S^{\prime }}^{n+1}}{\sigma_{eq}^{\prime }{}^{n+1}}\right]=\frac{1}{w^{n}}\sigma_{tr}^{n+1}-\frac{1}{w^{n+1}}C^{0}\Delta \Gamma \left[\frac{3}{2}\frac{{S^{\prime }}^{n+1}}{\sigma_{eq}^{\prime }{}^{n+1}}\right]$$

Using Equation (21) in (48), we get the simplified form for the iteration equation in indicial notation, as follows:

(49) $$\sigma_{eq}^{\prime }{{}^{n+1}}^{2}\left\{\delta_{ik}\delta_{jl}+\frac{1}{w^{n+1}}C_{ijkl}^{0}\Delta \Gamma \left[\frac{3}{2}\frac{1}{\sigma_{eq}^{\prime }{}^{n+1}}\right]\right\}\left\{\delta_{ik}\delta_{jl}+\frac{1}{w^{n+1}}C_{ijkl}^{0}\Delta \Gamma \left[\frac{3}{2}\frac{1}{\sigma_{eq}^{\prime }{}^{n+1}}\right]\right\}\;=\frac{\sigma_{eq}^{tr}{{}^{n+1}}^{2}}{w^{n}{}^{2}}\;$$

Algorithmically derived Equation (49) can be solved by an iteration procedure to find the value of ΔΓ, simultaneously with the update of w. A Newton-Raphson iteration scheme is employed in the integration scheme of the present study to solve the yield function given in Equation (32). Successively, the entropy is updated using Equation (27) and the damage is calculated using Equation (15).

## 3. Validation of the Computational Model for Monotonic Loading

Prior to the simulation of fully reversed cyclic loading, it is important to check the validity of the model under tensile as well as compressive loading. The computation models described in Section 2.3, is implemented in commercial finite element package, ABAQUS. User material subroutine is written to update the stresses according to the strain increments that are supplied to the subroutine as input. In order to validate the model for tensile as well as compressive loading cases in Ti-6Al-4V, we have used the experimental data, reported by Biswas et al. [2] and Carrion et al. [76].

### 3.1. Validation of the Numerical Model for Monotonic Tensile Loading

**Figure 3:**
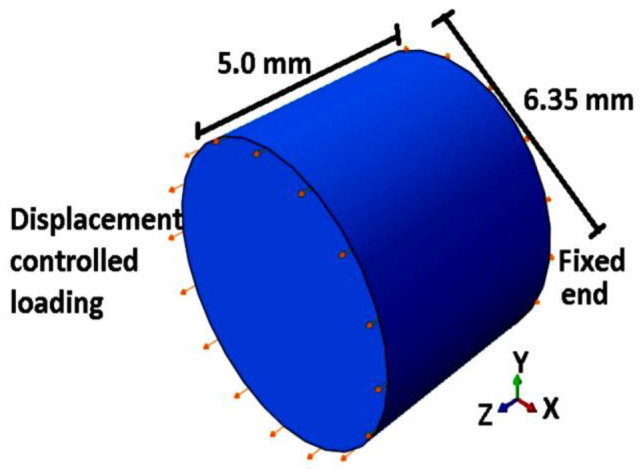
Schematics of numerical model for displacement controlled monotonic tensile loading in ABAQUS.

The true stress-strain graph reported in the literature [76] for Ti-6Al-4V alloy, is used for the comparison between experimental data and the numerical predictions of monotonic tensile loading. Mill Annealed hot rolled bars were used [76] in the study. The material parameters are taken from the literature [76], so as to match with the material used for the comparison. Details of the model parameters are given in Table 1. Using the common assumption that the gauge section of a dog bone sample experiences uniform strain, we consider 5 mm length in the computational model. Hence, it can reduce the computational cost as well. Diameter of the specimen is kept the same, like that of the experimentally reported sample by Carrion et al. [76], which is 6.35 mm in diameter. In ABAQUS, linear brick elements, C3D8R are used to mesh the numerical model. One end of the sample is defined with zero displacement (fixed) boundary condition and the other end is subjected to controlled displacement loading in the axial direction. After a mesh convergence analysis, an optimum seed size of 0.9 mm is fixed for all the simulations. A schematic representation of the computational geometry is shown in Figure 3. 

**Figure 4:**
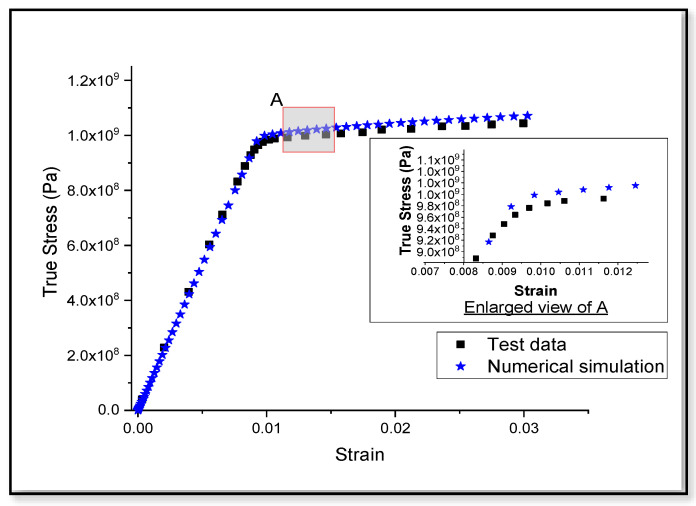
Comparison between monotonic tensile stress-strain graphs obtained from the test data [76] and numerical model.

It can be observed from the Figure 4 that the true stress-strain graph, predicted for monotonic tensile loading in Ti-6Al-4V alloy, matches well with the experimental observations reported by Carrion et al. [76]. A smooth transition can be seen at point A, shown in Figure 4. This transition from elastic to plastic region can be due to the dislocation motion in the microstructure. Further, dislocation multiplication and interaction with each other and inclusions can be the possible reason behind strain hardening of the bulk material. Hence, the validation of the model under tensile loading can be considered as a basis for tensile loading in any kind of geometry or boundary conditions in the numerical investigation.

**Figure 5:**
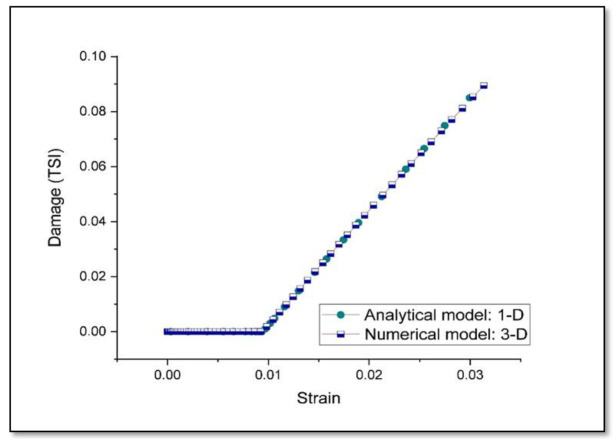
Comparison between the damage (TSI) prediction for monotonic tensile loading.

A comparative plot between the numerical results for damage obtained from three-dimensional model and analytical results based on one dimensional approximation, as described in Section 2.2, is shown in Figure 5. It is observed that the level of matching between computational and experimental results for monotonic tensile loading is closer in the case of prediction of damage, based on the analytical approach and numerical analysis. 

### 3.2. Validation of the 3-D Numerical Model for Monotonic Compressive Loading

**Figure 6:**
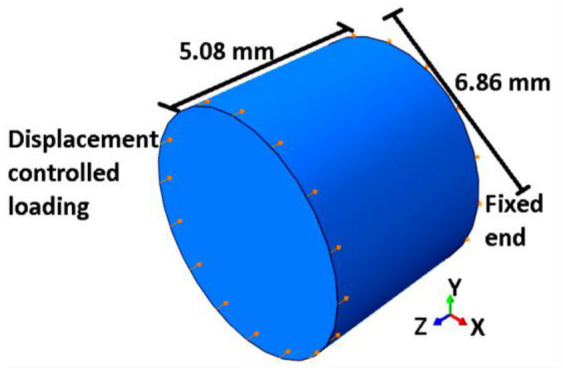
Schematics of numerical model for displacement controlled monotonic compressive. loading in ABAQUS.

Validation of the 3-D numerical model is done under compressive loading as well. Experimental result for a monotonic compression test, reported in the literature [2] is used to validate the proposed numerical model. The computational model parameters are taken from the literature [2], so as to match with the material used for the comparison. Even though the reported experimental results [2,76] are for Ti-6Al-4V alloys, it is noted that the materials are different in terms of their mechanical properties. Details of the model parameters used for the numerical simulation of monotonic compression test are listed in Table 2. We have considered the same dimensions in the numerical model, as that of the experimental samples [2]. Since, true stress-strain data is given in the literature [2], analytical procedure to compute TSI, requires an additional step. This method is adopted from well-known damage rule based on area reduction [77]. In the current study, damage parameter is represented by the TSI. Hence, the current area is related to the original area of undamaged section through the factor, TSI as follows:

(50) $$A=\left(1-\Phi \right)A_{0}$$

where, *A* and $A_{0}$ represents the current area and initial area. The engineering stress and true stress are related by the principle of static equilibrium as follows,

(51) $${\bar{\sigma }}^{\prime }A=\bar{\sigma }A_{0}$$

where, ${\bar{\sigma }}^{\prime }$ and $\bar{\sigma }$ represents the true stress and engineering stress respectively. Hence, in order to quantify the entropy, we have estimated the engineering yield stress data as follows:

(52) $${\bar{\sigma }}_{y}{}^{i+1}={\bar{\sigma }}_{y^{\prime }}{}^{i+1}\left(1-\Phi^{i}\right)$$

where, ${\bar{\sigma }}_{y}{}^{i+1}$ and ${\bar{\sigma }}_{y^{\prime }}{}^{i+1}$ represents the computed engineering stress and true stress at (*i*+1)-th strain, respectively. $\Phi^{i}$ is calculated based on the *i*-th strain data. Hence, in an incremental way, the TSI is computed using analytical procedure given in Section 2.2. Computation model in ABAQUS is discretized with linear brick finite elements C3D8R. One of the ends of the computational model is constrained from all the translations and the other end is subjected to displacement controlled compressive loading in the axial direction. A mesh convergence analysis is conducted and an optimum seed size of 0.9 mm is adopted in the simulations. A schematic representation of the computational geometry is shown in Figure 6.

**Figure 7:**
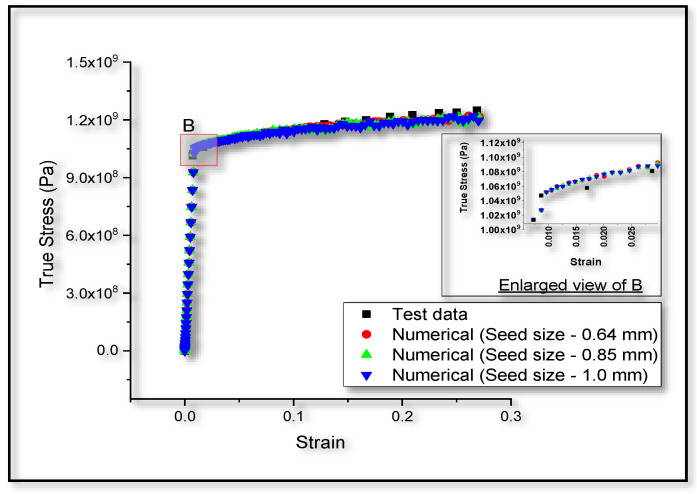
Comparison between monotonic compressive stress-strain graphs obtained from the test data [2] and numerical model.

**Figure 8:**
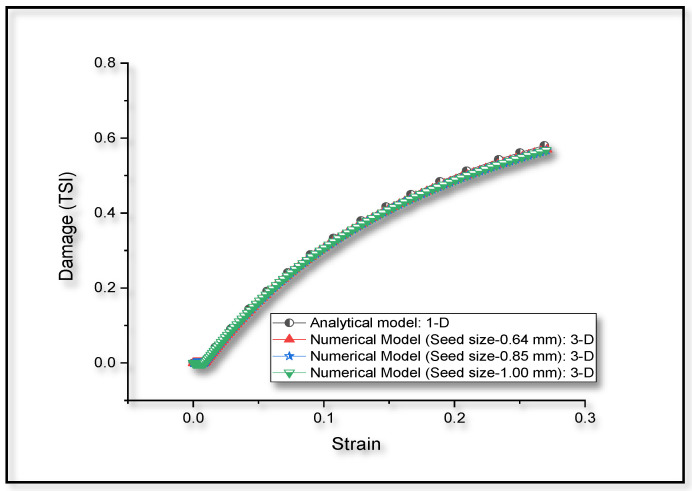
Comparison between the damage prediction for monotonic compressive loading.

Numerical results for monotonic compressive loading in Ti-6Al-4V alloy, shown in Figure 7, are found to be matching well with the reported experimental results [2]. Hence, the proposed model is taken as a basis to simulate compressive loading cases in the succeeding numerical investigations. Using the experimental [2] stress-strain graph, we have analytically calculated the TSI at every incremental plastic strain, based on the procedure stated in Section 2.2. As shown in Figure 8, both the analytical and numerical predictions for TSI matches very well.

## 4. Model Predictions for Low Cycle Fatigue Life 

Carrion et al. [76] tested Ti-6Al-4V samples under tensile loading condition at a strain rate of the order of 10^−3^ s^−1^ at room temperature. Similar quasi-static loading condition is established in our numerical loading by controlling the step time of the numerical model in ABAQUS. The material model used in developing the 3-Dimensional numerical model is independent of the strain rate and the temperature and hence the strain rate hardening behavior and temperature effects, including the thermal dissipation are not considered in our study. Unified mechanics theory-based approach for damage calculation, described in Section 2, is used to predict the low cycle fatigue life of Ti-6Al-4V alloys. Details of the one-dimensional analytical model as well as the three-dimensional numerical model to predict fatigue life or Ti-6Al-4V are given in Section 4.1 below.

### 4.1. Analytical Approach for Fatigue Life Prediction

**Figure 9:**
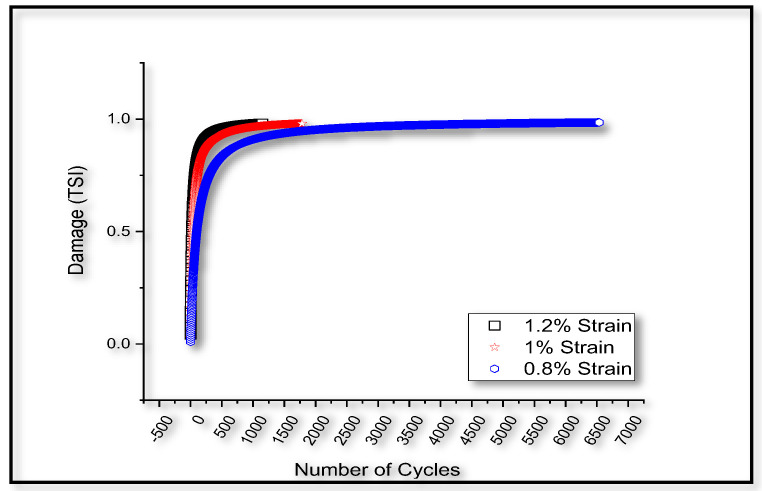
Analytical prediction of damage for different strain amplitudes of cyclic loading.

Experimental results [76] for the stabilized hysteresis loop is assumed to be closer to the first cycle hysteresis loop. Unified mechanics theory is used to evaluate the damage evolution under cyclic loading and the results are plotted in Figure 9.

**Figure 10:**
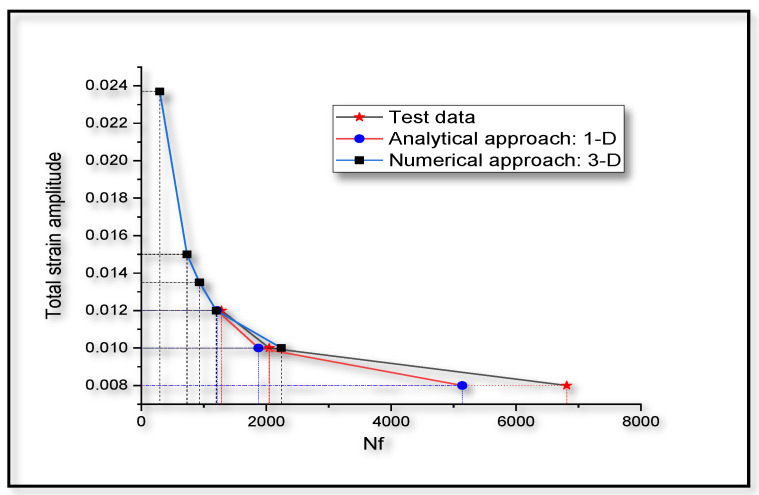
Low cycle fatigue life (Nf) prediction at different strain amplitudes of cyclic loading in comparison with the test data [76].

Low cycle fatigue life of the Ti-6Al-4V sample is predicted by fixing the TSI at failure as 0.98. This is necessary, as to prevent computational instabilities at the verge of failure that are not recorded by experimental results, are to be taken into account when we compare the mathematical model predictions with the experimental results. A MATLAB script is written to compute the fatigue life, from the stabilized hysteresis loop. The results are shown in comparison with the test data [76] and the corresponding numerical predictions at similar amplitudes, as shown in Figure 10.

### 4.2. Computational Procedure for Fatigue Life Prediction

It is not feasible to conduct a large number of cyclic loading in the numerical model to predict fatigue life, especially when the amplitude of strain is very small because in ABAQUS this process would take weeks. In this section, we propose an alternate way of fatigue life prediction of Ti-6Al-4V alloys at different strain amplitudes, using a combined numerical-experimental procedure. If the hysteresis loop for a given strain amplitude is found out from the experiment, the same test is simulated by using the proposed model. Computational results after the first cycle of loading are used to find the scaling factor for incremental entropy in the computational model. The scaling factor is calculated as the ratio between the experimental hysteresis loop area for the stabilized loop and the numerically computed dissipation for the first cycle of loading. Then the computational model is used to evaluate the dissipation at different strain amplitudes of loading for a single cycle of loading. This hysteresis loop is used to predict the fatigue life at the given amplitude of strain, as per the procedure detailed in Section 2.3. 

**Figure 11:**
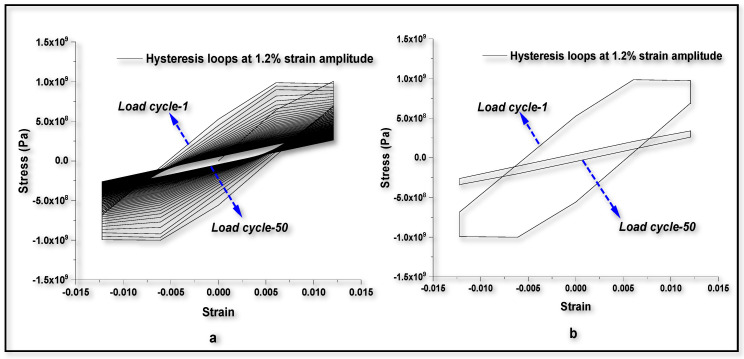
Numerical results on engineering stress-strain hysteresis loops for 1.2% strain amplitude of cyclic loading. (**a**) hysteresis loops at 1.2% strain amplitude for 50 cycles of loading; (**b**) comparative hysteresis plot for first cycle and 50th cycle of loading.

To compare the numerical predictions for fatigue life with experimental results [76], the same material data, as listed in Table 1, are used. It is assumed that the experimental results are free from any boundary effects or instabilities. Hence, the numerical analysis is done on the sample, with dimensions and boundary conditions as shown in Figure 3. Hysteresis loops at 1.2% strain amplitude for 50 cycles of loading are plotted in Figure 11a. A comparative hysteresis plot for first cycle and 50th cycle of loading is shown in Figure 11b. It can be observed from Figure 11a,b, that the hysteresis loop area decreases with cyclic loading. This reduction in hysteresis loop area is due to the reduction in strength of the material with the evolution of TSI. The fatigue life can be predicted by extrapolating the numerical results on TSI axis vs number of cycles. A comparison plot between test data and simulations for low cycle fatigue life prediction at different strain amplitudes is shown in Figure 10. In Figure 10, the average values of fatigue life test data [76] are plotted for stain amplitudes of 0.8%, 1.0% and 1.2% and compared with the analytical predictions. Fatigue life test data for other amplitudes of strain are not reported in the literature [76]. Results from the numerical approach, for the strain amplitudes 1.0% and 1.2% are also plotted and the model prediction is extended to a strain amplitude of 2.4%. Response at 0.8% strain amplitude was not computed with 3-D model because 0.8% strain amplitude is within the elastic region of loading.

In Figure 10, it is clear that the one-dimensional analytical approach is underestimating the fatigue life by 1600 cycles at a total strain amplitude of 0.8%, while this discrepancy is less scattered in the test data [76]. This discrepancy in fatigue life prediction using one-dimensional model could be due to the unaccounted three-dimensional confinement effects in material response.

## 5. Conclusions 

The work presented here is based on the unified mechanics theory, where the laws of Newtonian mechanics are combined with laws of thermodynamics, directly. The bridging factor in unified mechanics theory is the definition of thermodynamic state index, given in the Equation (9). The definition of damage proposed in the literature [29], is applied in the case of monotonic as well as low cycle fatigue loading conditions in Ti-6Al-4V alloys. Based on the principles of continuum mechanics, we have presented a numerical model, which account for the damage in case of plastic loading in Ti-6Al-4V. It is observed from the three-dimensional numerical and one-dimensional analytical results of the damage model prediction that, they match very well with the experimental observations in the case of monotonic tensile loading, as shown in Figure 4 and Figure 5. In Figure 4, we have considered the stress-strain graph given in the literature [76] for validation and the corresponding damage prediction (value of TSI is around 0.1) is limited to a strain level of about 3%. Linear extrapolation of the damage curves plotted in Figure 5, can lead to wrong prediction of failure strain (to around 20% in the current study). Entropy at each time increment is dependent on the stress level. Hence, the accuracy of life prediction will be dependent on the constitutive model, used to predict the yielding of the material, in a three-dimensional numerical study. 

The monotonic compressive stress-strain graph is matching well with the experimental results reported in the literature, as shown in Figure 7. The path traced by the damage prediction from one-dimensional analytical procedure and three-dimensional numerical procedure, as shown in Figure 8, also matches very well. In the case of compressive loading conditions, the results can be affected by the confining effects. The difference in nature of path traced by damage curves in compressive and tensile loading conditions could be due to the difference in confining effects in compressive loading, when compared with tensile loading. Similar observations for alloys can be seen in the literature [78,79]. Current study may be extended in future, for the detailed experimental and numerical investigations on such confining effects, under compressive loading. Since the current focus of the investigation is to introduce an efficient way of predicting the fatigue life of Ti-6Al-4V using computational tools in conjunction with the experiment, we have limited our study to fatigue life prediction.

Thermodynamics of life of any system, as postulated by the unified mechanics theory, is brought in to application level, for the case of low cycle fatigue life prediction in Ti-6Al-4V. From the comparative study on fatigue life prediction, as shown in Figure 10, the proposed procedures, described in Section 4, are found to be very efficient. Only one cycle experimental data is sufficient to predict the low cycle fatigue in Ti-6Al-4V alloys. Hence, the procedure stated in Section 4, will be useful for practical applications.

## Figures and Tables

**Table 1:** Material parameters used in the numerical model for tensile loading in Ti-6Al-4V alloy.

Material Parameter	Value	Unit
**Young’s modulus, E**	106	GPa
**Poisson’s ratio, ν**	0.31	
**Density,** ρ	4540	kg/m^3^
**Critical TSI,** $\Phi_{c}$	1	
**Hardening parameter,** K	968.00	MPa
**Hardening exponent,** r	0.64	
**Yield strength,** $\sigma_{y_{0}}$	992.00	MPa
**Molar mass,** $m_{s}$	0.047867	kg/mol
**Reference temperature, *T***	298	K

**Table 2:** Material parameters used in the numerical model for compressive loading in Ti-6Al-4V alloy.

Material Parameter	Value	Unit
**Young’s modulus, E**	118	GPa
**Poisson’s ratio, ν**	0.31	
**Density,** ρ	4540	kg/m^3^
**Critical TSI,** $\Phi_{c}$	1	
**Hardening parameter,** K	550.00	MPa
**Hardening exponent,** r	0.65	
**Yield strength,** $\sigma_{y_{0}}$	1047.00	MPa
**Molar mass,** $m_{s}$	0.047867	kg/mol
**Reference temperature, *T***	298	K
